# Identification of Predictors of Adaptability in Older Adults Based on the Roy Adaptation Model Using Machine Learning

**DOI:** 10.3390/jcm15051709

**Published:** 2026-02-24

**Authors:** Javier Gaviria Chavarro, Miguel Ángel Gómez García, Jose Manuel Alcaide Leyva, Alfonsina del Cristo Martínez Gutiérrez, Rosa Nury Zambrano Bermeo

**Affiliations:** 1 Doctoral Program in Applied Sciences, Faculty of Basic Sciences, Universidad Santiago de Cali, Cali 760011, Colombia; javier.gaviria@gmail.com; 2 Department of Basic Sciences, Institución Universitaria Escuela Nacional del Deporte, Cali 760033, Colombia; mangelgomez@endeporte.edu.co (M.Á.G.G.); adcmartinez@endeporte.edu.co (A.d.C.M.G.); 3 Departamento de Enfermería, Farmacología y Fisioterapia, Facultad de Medicina y Enfermería, University of Córdoba, 14014 Cordoba, Spain; n12allej@uco.es; 4 Nursing Program, Faculty of Health, Universidad Santiago de Cali, Cali 760011, Colombia

**Keywords:** Roy Adaptation Model, older adults, Senior Fitness Test, SF-12, WHO-5, machine learning, adaptation

## Abstract

**Background**: The Callista Roy Adaptation Model posits that adaptation in later life emerges from the interaction among physical, psychological, and social dimensions. However, empirical evidence integrating these domains through predictive approaches remains limited. The aim of this study was to identify the main predictors of adaptive classification in older adult women using functional and subjective well-being measures. **Methods**: A predictive study was conducted in older adult women enrolled in community-based exercise programs. Assessments included the Senior Fitness Test and the SF-12 and WHO-5 questionnaires. Multiclass classification models were trained, with Random Forest selected due to superior performance. Model evaluation incorporated oversampling strategies and robustness analyses without oversampling, using metrics resilient to class imbalance (macro-F1 and balanced accuracy). Model interpretability was examined through variable importance analysis, partial dependence, and ICE plots. **Results**: Under the oversampling framework, the Random Forest model achieved an overall accuracy of 74% and a macro-F1 score of 0.73, with reduced performance observed in robustness analyses, particularly for the minority “High” class. The most influential predictors were the physical component of the SF-12, the 2 min step test, the mental component of the SF-12, and the chair sit-and-reach test. **Conclusions**: The findings highlight the joint contribution of physical and psychosocial factors to adaptive processes, in alignment with the Roy Adaptation Model. This study provides exploratory evidence supporting the integrated use of the SFT, SF-12, and WHO-5; however, external validation and longitudinal evaluation are required prior to clinical implementation.

## 1. Introduction

Human development encompasses different stages, from childhood to older adulthood, during which declines in muscle mass, bone density, and flexibility commonly occur [1,2]. These changes can negatively affect health and autonomy, underscoring the need for programs that strengthen adaptive capacity. The Roy Adaptation Model conceptualizes the person as an adaptive system in which human beings are multidimensional entities composed of biological, psychological, and social subsystems. Because these dimensions interact and shape health and quality of life, optimizing physical and psychological health is essential to preserve functional independence and overall well-being in later life [3,4].

Adaptation in later life constitutes a dynamic and multidimensional process that cannot be understood solely in terms of the absence of disease, but rather as the result of the continuous interaction among physical, psychological, and social systems. In this regard, Callista Roy’s Adaptation Model conceptualizes the individual as an adaptive system capable of responding to internal and external stimuli through coping processes that are expressed in observable patterns of effective adaptation or adaptive inefficacy. Recent reviews have shown that, although Roy’s model has been widely used to guide interventions in older adults, much of the empirical evidence has focused on descriptive approaches or isolated domains, thereby limiting the identification of factors that explain interindividual variability in adaptive capacity during aging [4,5]. This limitation underscores the need for integrative approaches that allow joint analysis of functional and psychosocial determinants of adaptation.

From a functional perspective, instruments such as the Senior Fitness Test (SFT) are designed to assess functional fitness in older adults through a battery of standardized tests, including the chair stand test, arm curl test, 6 min walk test, 2 min step test, chair sit-and-reach test, back scratch test, and the 8-foot up-and-go test [6]. Psychological well-being and health-related quality of life can be assessed using validated questionnaires such as the WHO-5 and the SF-12, which capture subjective well-being, mood, and perceived health status [7,8]. Normative studies and systematic reviews have demonstrated that performance on SFT components is associated with clinically relevant outcomes such as fall risk, functional decline, and loss of independence, confirming its utility as a tool for characterizing functional trajectories in aging [8,9,10].

In this context, the integration of functional and psychosocial measures through predictive approaches represents a methodological opportunity to advance the understanding of adaptive processes in older adults. However, the use of machine learning models in health research requires a rigorous framework of transparency, interpretability, and methodological validity, particularly when the aim is to identify predictors with potential clinical relevance. The TRIPOD+AI guidelines, recently published in The BMJ, emphasize the importance of comprehensive reporting of model development, validation, and evaluation procedures, as well as strategies for handling class imbalance and interpreting results, in order to avoid optimistic or non-reproducible inferences [11]. In line with these recommendations, integrating Roy’s Adaptation Model with functional indicators derived from the SFT and measures of subjective well-being, such as the SF-12 and WHO-5, through interpretable predictive models allows for a systematic exploration of the determinants of adaptation in later life, while maintaining an analytical approach consistent with contemporary standards in health research.

Complementarily, instruments such as the SF-12 and the WHO-5 capture subjective dimensions of physical health, mental health, and psychological well-being that reflect the lived experience of aging and have shown sensitivity to changes associated with exercise-based and socially oriented interventions [7,12].

Research applying Roy’s Adaptation Model in older adults has often focused on adaptation to specific health-related circumstances, with less emphasis on integrated functional and psychosocial assessment across this life stage. In this context, the SFT and well-being scales allow researchers to evaluate both physical and mental status, thereby complementing Roy’s framework [6,10,13]. However, studies that simultaneously integrate the Roy Adaptation Model, the SFT, and instruments such as the WHO-5 and SF-12 within the same sample of older adults remain scarce. Because each instrument captures distinct functional, psychological, and adaptive dimensions, their combined use can support the design of exercise programs tailored to individual needs [4,7,10].

In the Latin American context, it is essential to investigate which variables promote adaptive responses among older adults, given the projected increase in this population group [14]. This demographic transition encourages research on aging and on programs aimed at maintaining and improving the quality of life. Autonomy in older adults has been associated with physical, social, and psychological dimensions; therefore, when social interaction or functional independence declines, perceived quality of life may deteriorate [5].

The Roy Adaptation Model provides a framework for studying adaptive responses and guiding interventions aimed at improving quality of life [5,13]. The SFT also contributes to identifying musculoskeletal impairments such as sarcopenia and limitations in agility, flexibility, and coordination [10,15]. In addition, questionnaires such as the WHO-5 and SF-12 provide indicators of psychological well-being and perceived health status in older adults [12]. Accordingly, the objective of this study was to identify key predictors of adaptive classification under Roy’s Adaptation Model by integrating SFT outcomes with WHO-5 and SF-12 measures in older adults.

## 2. Materials and Methods

### 2.1. Study Design

The research was conducted using a quantitative approach within a prospective longitudinal framework and employed a non-randomized intervention design. Assessments were performed at two predefined time points: prior to the implementation of the intervention and upon its completion. Both baseline and post-intervention evaluations were carried out by trained professionals in nursing and physical activity, using validated psychological and physical assessment batteries. This longitudinal assessment strategy enabled the examination of changes over time and the identification of key predictors of adaptive responses as conceptualized in Roy’s Adaptation Model. The absence of random allocation to intervention groups situates the study within the domain of quasi-experimental research designs.

### 2.2. Population and Sample

The study population comprised community-dwelling older adults who regularly attended structured physical exercise programs offered at public sports and recreational facilities. Individuals were eligible for inclusion if they were aged 60 years or older, demonstrated preserved cognitive functioning, were able to communicate effectively with the research team, and had sufficient functional autonomy to ambulate and attend exercise sessions independently. Participation was voluntary, and all eligible individuals provided written informed consent prior to enrollment.

Participants were excluded if they presented with acute traumatic injuries; a recent history of musculoskeletal disorders within the preceding six months; surgical procedures performed or scheduled within three months before or after study initiation; uncontrolled cardiac arrhythmias; or inadequately controlled non-communicable diseases, including hypertension or diabetes mellitus. Additional exclusion criteria included documented medical contraindications to physical activity, the use of orthotic devices, concurrent participation in other structured exercise programs, or failure to attend at least 85% of the sessions prescribed in the intervention protocol.

Sample size estimation was conducted using G*Power software (version 3.1.7), considering a non-randomized design with three intervention groups, a 95% confidence level, an alpha error of 0.05, a statistical power of 0.90, and an expected effect size of 0.32. The calculation yielded a minimum required sample of 407 participants. To compensate for potential losses during follow-up, a 10% attrition rate was anticipated, resulting in a final target sample of 450 older adults, with 150 participants assigned to each intervention group.

### 2.3. Instruments

#### 2.3.1. Dependent Variable (Clasf_Roy)

The primary outcome of the study was the classification of participants’ coping and adaptation capacity, grounded in Roy’s Adaptation Model. This construct was operationalized using the abbreviated and culturally adapted Spanish version of the Coping and Adaptation Processing Scale (CAPS/EsCAPS), which comprises 33 items [16]. Responses are recorded on a four-point Likert scale ranging from 1 (never) to 4 (always), yielding a total score between 33 and 132, with higher scores indicating greater adaptive and coping capacity.

For analytical purposes, an ordinal outcome variable (Clasf_Roy) was derived by categorizing the total score into three levels according to cut-off points previously reported for this abbreviated version: low (33–89), moderate (90–103), and high (104–132) adaptive coping capacity [17].

SFT. The Senior Fitness Test (SFT) is a standardized battery designed to assess functional fitness in older adults. It provides an integrated evaluation of physical capacities relevant to independent functioning, including muscular strength, flexibility, agility, and dynamic balance of both the upper and lower extremities. In the present study, the core components of the SFT were administered. These included the chair stand test (lower-body strength; number of repetitions completed in 30 s), the arm curl test (upper-body strength; number of repetitions in 30 s), the 2-Minute Step Test (aerobic endurance; number of knee raises reaching a predetermined point located midway between the patella and the iliac crest during a 2 min period), the chair sit-and-reach test (lower-body flexibility; distance measured in centimeters), the back scratch test (shoulder flexibility; distance in centimeters), and the 8-foot up-and-go test (agility and dynamic balance; time recorded in seconds) [8].

#### 2.3.2. SF-12 Health Survey

The 12-Item Short Form Health Survey (SF-12) is a self-administered instrument used to assess health-related quality of life. It comprises 12 items that capture eight domains of physical and mental health, which are summarized into the Physical Component Summary (PCS) and the Mental Component Summary (MCS). These domains include general health, physical functioning, role limitations due to physical problems, bodily pain, vitality, social functioning, and role limitations due to emotional problems [18].

#### 2.3.3. WHO-5 Well-Being Index

The World Health Organization Five Well-Being Index (WHO-5) is a brief self-report measure consisting of five items that assess subjective psychological well-being, focusing on vitality, positive mood, and interest in daily activities. Responses are recorded using a Likert-type scale [19,20].

### 2.4. Statistical Analysis

Multiclass machine learning methods were applied to a dataset comprising 450 older adult women (150 per intervention group), assessed at baseline and after completion of the intervention. Prior to model development, data completeness was systematically examined. No missing values were identified in any of the variables included in the analysis; consequently, no data imputation procedures were required.

Predictor variables were operationalized as change scores, calculated as the difference between post-intervention and baseline values. These predictors encompassed indicators of functional physical fitness derived from the Senior Fitness Test battery, measures of psychological well-being and health-related quality of life (WHO-5 and SF-12 PCS/MCS), intervention group, and age category.

The analytic dataset was constructed at the participant level (one record per participant). Predictors were defined as pre–post change scores (Δ = post − baseline). The outcome (Clasf_Roy) was derived exclusively from the post-intervention abbreviated EsCAPS total score (range: 33–132) and categorized into low (33–89), moderate (90–103), and high (104–132) levels.

To avoid information leakage, the dataset was first split into training (70%) and test (30%) sets using stratified sampling based on the multiclass outcome (Clasf_Roy). The test set was kept fully untouched throughout model development. All preprocessing steps (z-score standardization and, when applicable, SMOTE oversampling) were fitted using training data only. Specifically, standardization parameters were estimated on the training subset and applied to the corresponding validation/test partitions only after fitting. During stratified 5-fold cross-validation, SMOTE was performed exclusively within each training fold, ensuring that synthetically generated samples did not influence validation folds or the held-out test set.

Three multiclass classification algorithms were evaluated: multinomial logistic regression, Random Forest, and Extreme Gradient Boosting (XGBoost). Model performance was assessed using stratified five-fold cross-validation, with the macro-averaged F1-score selected as the primary metric for model comparison to account for class imbalance.

These modeling approaches were selected to represent complementary families of classifiers with distinct assumptions and representational capacities, enabling examination of whether the relationship between pre–post changes (Δ) in functional status, well-being, and quality-of-life measures and adaptive classification (Clasf_Roy) could be adequately captured by approximately linear effects or required modeling of nonlinear patterns and higher-order interactions. Multinomial logistic regression was included as a reference model due to its probabilistic framework, interpretability, and stability in moderate sample sizes, allowing estimation of the direction and relative magnitude of associations through predicted probabilities. Random Forest was selected for its capacity to model nonlinear relationships and complex interactions without prior specification, as well as its robustness to multicollinearity among predictors. XGBoost was incorporated as a high-performance gradient boosting approach for tabular data, capable of capturing complex nonlinear structures, thereby allowing assessment of whether increased model flexibility enhanced discriminatory performance relative to linear modeling. Together, this strategy balanced interpretability and predictive power, aligning model selection with the objective of multiclass prediction of adaptive status based on change scores.

Given the imbalance across outcome categories, imbalance-aware learning strategies were examined. In the primary analysis, synthetic oversampling was implemented using the Synthetic Minority Oversampling Technique (SMOTE), which generates minority-class instances through interpolation in the predictor space [21]. To prevent information leakage, the dataset was initially partitioned into training (70%) and testing (30%) subsets using stratified sampling. The test set was preserved without modification, while standardization and oversampling procedures were fitted exclusively on the training data and applied only within the corresponding folds or partitions.

During model training, predictor standardization and oversampling (when applied) were implemented within a unified pipeline restricted to the training data, and in cross-validation, exclusively within each training fold, thereby ensuring that synthetically generated observations did not influence model evaluation [22]. Furthermore, given that predictor variables consisted of change scores (Δ = post − pre), it was acknowledged that oversampling might generate synthetic combinations that are mathematically plausible but not necessarily physiologically realistic. Consequently, sensitivity analyses were conducted without oversampling, using cost-sensitive learning approaches with class weighting (class_weight) to evaluate the robustness of model performance [23,24].

The model demonstrating the highest performance was subsequently subjected to hyperparameter optimization using grid search (GridSearchCV), with the macro-averaged F1-score as the optimization criterion. Final model performance was evaluated on the held-out test set using standard classification metrics (precision, recall, and F1-score), confusion matrices, and class-specific Receiver Operating Characteristic (ROC) curve analyses, including computation of the area under the curve (AUC) for each outcome category. Predictor importance was quantified using the impurity-based importance criterion derived from the Random Forest model. Additional interpretability analyses were performed using partial dependence plots to estimate the marginal effects of selected continuous predictors (Physical Component Summary score and 2-Minute Step Test performance) on the probability of classification into the “High” adaptive category.

All statistical analyses were conducted using Python version 3.10, with implementation based on the scikit-learn, xgboost, imbalanced-learn, matplotlib, seaborn, and pandas libraries.

### 2.5. Ethical Considerations

The study protocol was reviewed and approved by a health research ethics committee, which granted full ethical approval as documented in the official session minutes dated August 11, 2023, under Administrative Resolution No. 08 CFS 025-2007. The study was classified as minimal risk in accordance with the criteria established in Article 11 of Resolution 8430 of 1993.

All participants received clear and comprehensive information regarding the study objectives, procedures, and potential implications, and enrollment occurred only after written informed consent was obtained. Participant confidentiality and data anonymity were ensured through systematic data coding and the exclusive use of the information for scientific purposes, in accordance with the ethical principles governing research involving human participants.

## 3. Results

Participants were recruited from structured community-based exercise programs delivered at public sports and recreational facilities in Cali, Valle del Cauca, Colombia. Therefore, the study sample represents older adults who are sufficiently autonomous to attend organized exercise sessions and consent to assessments, rather than the general older-adult population. Consequently, external validity is primarily limited to community-dwelling older women participating in similar public exercise programs and socioeconomic contexts.

The population participating in the programs consisted predominantly of older adults aged 60–64 years aged 60–64 years, who represented the largest proportion of attendees (33.8%, *n* = 152). As age increased, a progressive decline in participation was observed: 18.9% (*n* = 85) were aged 65–69 years, 17.6% (*n* = 79) were between 70 and 74 years, and 13.3% (*n* = 60) were aged 75–79 years. The oldest age groups, although present, were less represented, with participants aged 80–84 years accounting for 9.1% (*n* = 41) and those aged 85–89 years accounting for 7.3% (*n* = 33).

From a socioeconomic perspective, the sample was largely homogeneous, with the vast majority of participants classified within socioeconomic stratum 3 (97.8%, *n* = 440), while only a small proportion belonged to stratum 2 (2.2%, *n* = 10). Regarding educational attainment, a substantial proportion reported having incomplete secondary education (44.2%, *n* = 199), whereas 36.2% (*n* = 163) had completed secondary education. Smaller proportions reported primary education only (13.6%, *n* = 61) or incomplete primary education (6.0%, *n* = 27), reflecting heterogeneous educational trajectories across the life course. In terms of marital status, married participants predominated (59.3%, *n* = 267), followed by widowed (21.3%, *n* = 96) and single individuals (19.3%, *n* = 87). With respect to occupational status, 40.4% (*n* = 182) identified as homemakers, 30.7% (*n* = 138) reported being currently employed, and 28.9% (*n* = 130) indicated that they were retired, as shown in Table 1 for these variables, and for the remaining predictor variables.

To identify the most relevant predictors of post-intervention adaptive classification according to Roy’s Adaptation Model (low, moderate, and high levels), three machine learning models—Random Forest, XGBoost, and multinomial logistic regression—were trained and compared. Predictor variables consisted of pre–post change scores (Δ = post − pre) in physical performance measures derived from the Senior Fitness Test, perceived well-being and quality-of-life indicators (PCS, MCS, and WHO-5), as well as age category and type of intervention received. Because the predictors reflected changes observed after the intervention, this approach does not constitute a prospective pre-intervention prediction model; rather, it represents an associative analysis aimed at identifying which concurrent changes were most strongly related to the adaptive classification observed following program completion.

Within the training subset (70%), while keeping the test set (30%) intact, SMOTE was applied exclusively during model training and restricted to the training folds within cross-validation, in order to address class imbalance without introducing information leakage. Under this framework, the model achieving the highest average performance in terms of macro-averaged F1-score was the Random Forest classifier (mean macro-F1 = 0.73). This model was subsequently fine-tuned using grid search, yielding the following optimal hyperparameters: number of trees = 100, unrestricted tree depth, minimum samples per split = 2, and minimum samples per leaf = 1.

In the final evaluation on the independent test set, the optimized Random Forest model achieved an overall accuracy of 74% and a macro-averaged F1-score of 0.73. Performance for the “High” adaptive classification was notably strong (precision = 0.92, recall = 1.00, F1 = 0.96). However, given that this category represented the least prevalent class, and acknowledging the inherent instability of performance estimates for rare outcomes, these results were interpreted cautiously and in conjunction with robustness analyses, including leakage-free pipelines and sensitivity analyses without oversampling, as well as with imbalance-robust metrics such as macro-F1, balanced accuracy, and the Matthews correlation coefficient (MCC). In contrast, the “Low” and “Moderate” classes showed moderate classification performance (F1 = 0.65 and 0.58, respectively), with a particularly reduced ability to correctly identify instances belonging to the “Moderate” category (recall = 0.53).

Sensitivity analyses conducted without synthetic oversampling, using cost-sensitive learning through class weighting, revealed a marked reduction in performance relative to the SMOTE-based scenario, particularly for the minority “High” class. In multinomial logistic regression with class weighting, overall accuracy declined to 0.51, with a macro-F1 of approximately 0.39 and balanced accuracy of 0.42 (MCC ≈ 0.16). Similarly, class-weighted Random Forest models yielded an accuracy of 0.59, macro-F1 of approximately 0.35, and balanced accuracy of 0.37 (MCC ≈ 0.11). These findings indicate that part of the performance gains observed under oversampling conditions may not generalize to settings without synthetic data generation. Accordingly, the model should be interpreted as exploratory in nature, particularly regarding discrimination of the infrequent “High” adaptive category.

**Figure 1:**
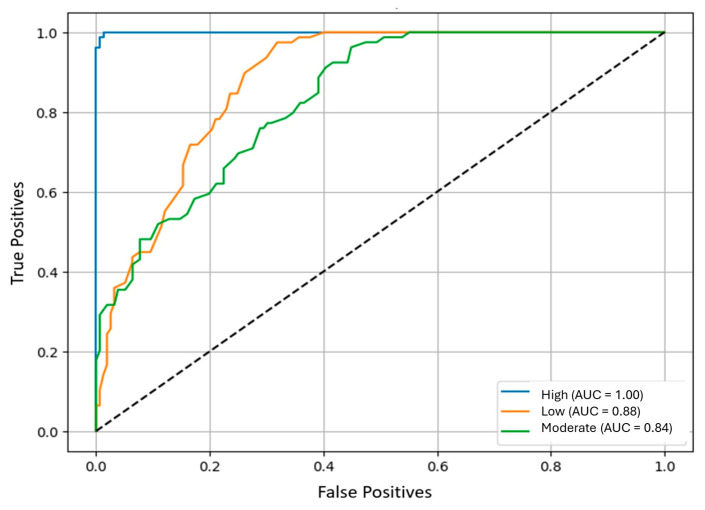
Class-specific ROC curves. Each curve reflects the model’s discriminatory ability for a specific class (Low, Moderate, or High). The horizontal axis represents 1 − specificity (false positive rate), while the vertical axis represents sensitivity (true positive rate), evaluated across all possible decision thresholds.

In the initial analysis, the “High” class exhibited an area under the ROC curve (AUC) of 1.00, Low and Moderate (0.88 and 0.84) under a one-vs-rest approach (Figure 1). Nevertheless, given the low prevalence of this class (“High”) and the use of oversampling, this result was interpreted with caution, as it is compatible with overfitting and/or information leakage when resampling procedures are not strictly confined to the training data. For this reason, the evaluation was repeated using a leakage-free pipeline, in which scaling and SMOTE were applied exclusively within training folds and the test set remained fully isolated, alongside a sensitivity analysis without oversampling based on class weighting. Under these more conservative conditions, performance for the “High” class proved substantially less stable, consistent with its low prevalence. Consequently, class-specific AUC values were considered exploratory, and greater emphasis was placed on imbalance-robust global metrics (macro-F1, balanced accuracy, and MCC) derived from the sensitivity analyses.

**Figure 2:**
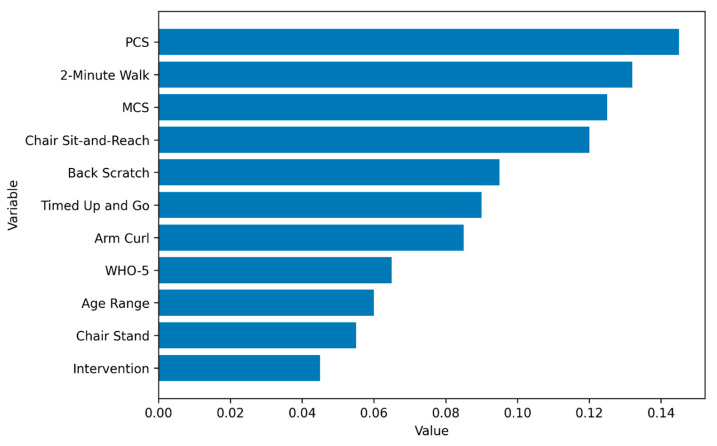
Feature importance analysis. The relative contribution of each predictor to model performance was estimated using the mean decrease in impurity, an internal criterion of the Random Forest algorithm, aggregated across all trees in the ensemble.

Variable importance analysis indicated that the most influential predictors of adaptive classification were the physical component of the SF-12 (PCS), performance on the 2-Minute Step Test, the mental component of the SF-12 (MCS), and the chair sit-and-reach test. These functional and self-perceived health indicators accounted for a substantial proportion of the observed variability in adaptive outcomes, exceeding the contribution of contextual variables such as intervention type or age group (Figure 2).

To further enhance the interpretability of the Random Forest model trained to predict adaptive classification according to Roy’s Adaptation Model, partial dependence plots (PDPs) were generated for PCS and the 2-Minute Step Test, identified as the most influential predictors based on variable importance rankings.

From a clinical and programmatic standpoint, the strongest predictors identified in this study (SF-12 PCS and MCS, the 2-Minute Step Test, and flexibility measures) are routinely used in community-based geriatric exercise settings and can be directly interpreted as actionable targets for intervention planning and monitoring. In practical terms, improvements in perceived physical health (PCS) and functional aerobic capacity (2-Minute Step Test), together with mental health and social functioning (MCS), suggest that favorable adaptation after exercise programs is more likely when physical capacity and subjective well-being improve in parallel. Therefore, these results support multicomponent strategies that integrate strength/mobility and aerobic training with approaches that foster psychosocial support and engagement. Importantly, because predictors were operationalized as change scores (Δ = post − pre), the model should be interpreted as identifying patterns of co-occurring improvements associated with adaptive classification at program completion, rather than as a pre-intervention prognostic tool for individual clinical decision-making.

**Figure 3:**
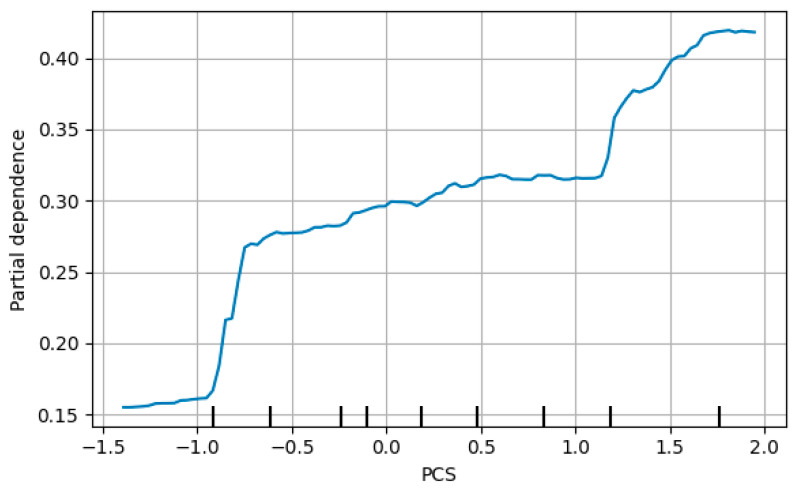
Partial dependence plot–PCS (class:High). The partial dependence plot (PDP) depicts the average marginal effect of PCS on the predicted probability of belonging to the “High” Clasf_Roy category, while the remaining predictors are held at their observed data distribution.

**Figure 4:**
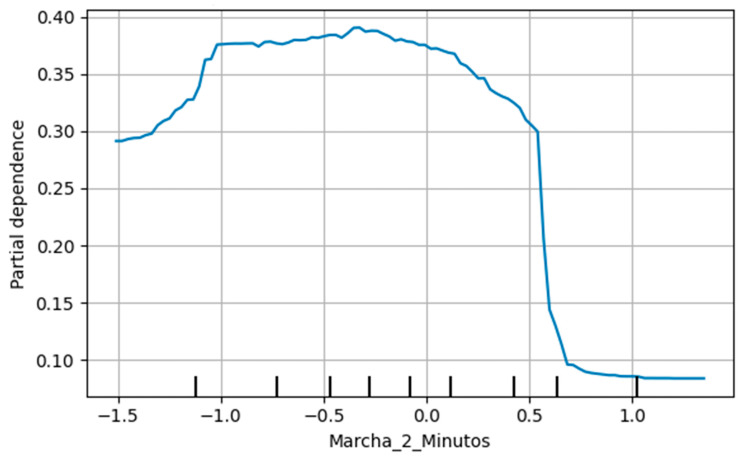
Partial dependence plot–2-minute step test (class: High). This variable represents the pre–post change (Δ = post − pre) in the number of knee raises completed during the 2-minute step test, following the protocol in which knee height is set at the midpoint between the patella and the iliac crest. For model training, all predictors were standardized using z-scores; accordingly, the x-axis is expressed in standardized units.

The PDP for PCS revealed a monotonic positive association between increases in self-reported physical health and the predicted probability of belonging to the “High” adaptive category. Specifically, beyond standardized values of approximately −1.0, a sustained upward trend was observed, with partial dependence exceeding 0.40 at standardized values approaching +2. This pattern suggests that better perceived physical health is consistently associated with a higher likelihood of favorable adaptive classification following the intervention (Figure 3). In contrast, the 2-Minute Step Test exhibited a nonlinear pattern (Figure 4).

**Figure 5:**
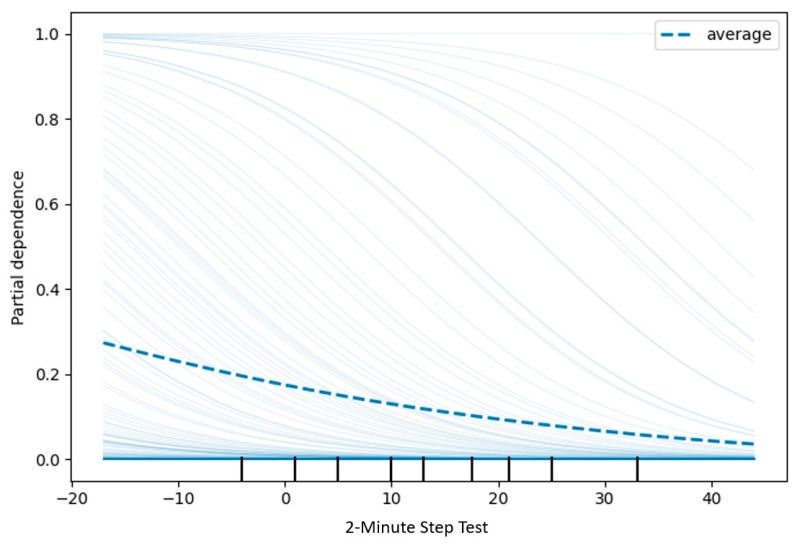
ICE + PDP 2-minute step test. The dashed line represents the PDP (average effect), whereas the individual lines correspond to ICE curves (Individual Conditional Expectation), which allow assessment of heterogeneity and potential interactions that are not captured by the average marginal effect.

Given that PDPs represent average marginal effects, their interpretation was complemented with Individual Conditional Expectation (ICE) curves to assess heterogeneity and potential interactions (Figure 5). While the average PDP suggested a slight decreasing trend in the predicted probability of the “High” category, ICE curves revealed substantial inter-individual variability in both the direction and magnitude of effects. Accordingly, this finding was interpreted as descriptive evidence of nonlinearity and/or interaction effects within the model, rather than as confirmation of a direct physiological relationship or a definitive functional threshold.

## 4. Discussion

The aim of this study was to identify the main predictors of adaptive classification according to Roy’s Adaptation Model. Under the oversampling framework, the Random Forest classifier achieved an overall accuracy of 74% and a macro-averaged F1-score of 0.73, indicating moderate predictive performance. However, performance declined in robustness analyses conducted without oversampling, particularly for the minority “High” adaptation class.

Although the oversampling-based analysis yielded a near-perfect one-vs-rest AUC for the “High” class, this result was interpreted with caution. The combination of (i) marked class imbalance and (ii) resampling strategies is known to generate optimistic or unstable estimates when not strictly confined to the training process. Consistent with this concern, robustness analyses, including a leakage-free pipeline and sensitivity analyses without oversampling, showed reduced performance for the “High” class. Accordingly, the model should be regarded as exploratory for this category, and greater emphasis was placed on metrics robust to class imbalance, such as macro-F1, balanced accuracy, and the Matthews correlation coefficient.

Variable importance analysis indicated that the most influential predictors of adaptive classification were the physical component of the SF-12 (PCS), performance on the 2 min step test, the mental component of the SF-12 (MCS), and the chair sit-and-reach test. These findings highlight the combined relevance of perceived health status and functional capacity in explaining adaptive outcomes beyond intervention group assignment or age range.

The prominence of psychological factors in the “High” adaptation class is consistent with prior evidence emphasizing the role of social relationships in later life, a dimension explicitly represented within the MCS domain [25]. During older adulthood, social interaction often decreases, particularly among individuals residing in geriatric institutions or those whose household roles have diminished. In this context, Roy’s Adaptation Model underscores strategies aimed at strengthening interpersonal relationships to enhance self-sufficiency and foster a sense of purpose through community engagement [3]. Other studies have shown that older adults living with younger family members may feel constrained in participating in daily activities, which can negatively affect self-esteem and perceived well-being [26]. Importantly, integrating Roy’s model with structured exercise programs has been shown to promote enjoyment of physical activity in older adults, with social interaction acting as a key mediator in improving perceived fulfillment and quality of life [27].

Similarly, the relevance of physical factors in older adults’ capacity to adapt to age-related changes has been widely documented. Attributes such as grip strength, gait speed, and the ability to maintain balance in standing positions have been identified as predictors of the need for physical activity interventions [28]. In the present study, the 2 min step test emerged as an important predictor; however, its partial dependence pattern was nonlinear and must be interpreted cautiously. Average marginal effects derived from PDPs do not, by themselves, allow the identification of definitive functional thresholds. Consequently, this finding is discussed as indicative of a complex relationship between functional aerobic capacity and adaptability rather than evidence of a specific physiological cutoff. It is plausible that, at advanced ages, fall risk increases and mobility-related demands become less central to adaptive processes as conceptualized by Roy’s model [9,29]. In line with previous studies, physical factors contribute substantially to subjective well-being and life satisfaction in older adults, through reductions in fatigue and improvements in functional capacity; therefore, integrating physical and psychological assessments supports a more comprehensive functional diagnostic framework [14].

Trunk flexibility also emerged as a relevant element for adaptability in older adulthood. Prior research has examined the association between spinal extensor and flexor strength and physical activity, showing that extensor strength is strongly related to activities of daily living, while trunk flexion has been linked to gait speed [30]. Additionally, Schönau and Anders (2023) [31], demonstrated that trunk flexor muscles play a role in spinal stability through intra-abdominal pressure during strength and resistance training. In seated postures, trunk flexors are essential for transitioning to standing and initiating gait, acting as stabilizers during movement; thus, assessments of adaptability in older adults should incorporate measures of trunk flexibility [32].

From a methodological standpoint, this study contributes an integrative approach by combining functional measures (Senior Fitness Test) and well-being and quality-of-life indicators (SF-12, WHO-5) within a unified analytical framework to model adaptive classification based on Roy’s Adaptation Model. While previous research has often examined physical or psychosocial components separately, the simultaneous integration of both domains through predictive modeling represents a promising direction. Nevertheless, this contribution should be interpreted within the exploratory nature of the study and requires replication and external validation.

The external validity of the findings is limited. The sample consisted exclusively of older women engaged in community-based exercise programs and exhibited high socioeconomic homogeneity, with a near-total predominance of a single socioeconomic stratum. This restricts the generalizability of the observed predictive patterns to other populations, including men and individuals from different socioeconomic, cultural, and geographic contexts. Such limitations are particularly relevant in predictive modeling, as changes in predictor distributions, outcome prevalence, and predictor–outcome relationships can substantially affect model performance. Therefore, before broader application, external validation in independent and more heterogeneous samples is required, potentially accompanied by model recalibration.

Additionally, the follow-up period was limited to 12 weeks, which may have constrained the detection of changes in certain outcomes and underestimated effects that require longer exposure. Future studies should incorporate longer follow-up periods to assess the stability of predictors and the persistence of adaptive changes.

Although the present findings provide exploratory evidence regarding predictors of adaptability in this cohort, translating a predictive model into decision-support tools in health care requires further steps in validation and governance. Generalizability must be demonstrated through external validation in independent samples incorporating variability by sex, socioeconomic status, territory, and implementation context, as well as through evaluation of calibration and clinical utility, not solely discrimination. In this regard, future work should adhere to contemporary recommendations for transparency and reporting of machine learning models in health research, such as TRIPOD+AI [11]. Additionally, MLOps frameworks have emerged to manage large volumes of health data and operationalize them through models, with the aim of reducing the time required to support clinical interventions, diagnosis, and patient treatment [33]. These frameworks have helped standardize how data should be handled across the lifecycle of machine learning solutions, encompassing data preparation, model training, model assessment and validation, deployment of model functionalities, as well as ongoing maturation, learning, and continuous monitoring [34,35]. Together, these components outline the key steps for implementing artificial intelligence in health care. Therefore, future research should incorporate MLOps frameworks to strengthen analytic rigor and to evaluate how models perform in dynamic, real-world settings.

Overall, the findings support the utility of Callista Roy’s Adaptation Model as a conceptual framework for understanding adaptive processes during aging through the integration of psychosocial and physical dimensions. Moreover, the model reinforces the relevance of instruments such as the Senior Fitness Test and psychological well-being questionnaires as tools not only for assessment but also for exploring adaptive capacity in older populations. Nevertheless, the results should be viewed as an initial methodological step; progression toward clinical application requires external validation, prospective evaluation, and an explicit strategy for model governance and performance monitoring over time.

## 5. Conclusions

From a practical perspective, the results of this study suggest the importance of targeted interventions aimed at improving physical conditions in older adults, particularly those related to mobility and strength, together with strategies that foster effective adaptive processes. In addition, approaches such as multicomponent exercise programs and psychosocial support are highlighted as relevant strategies to optimize adaptive responses in older adults, in alignment with Roy’s Adaptation Model.

Nevertheless, the application of the model as a decision-support tool in clinical practice requires further validation stages. These include external validation in more heterogeneous populations, assessment of model calibration and clinical utility, and the implementation of a systematic performance-monitoring plan to mitigate model drift and to ensure safety, equity, and applicability throughout the model’s lifecycle.

Finally, the findings of this study indicate that, from the perspective of Roy’s Adaptation Model, the Senior Fitness Test, SF-12, and WHO-5 are feasible instruments for assessing physical functioning, subjective well-being, and quality of life in later life. Accordingly, their use by health professionals, including nurses, physiotherapists, and other allied health practitioners, is appropriate for estimating and monitoring the functional and adaptive trajectories of older adults in both community and clinical settings.

## Figures and Tables

**Table 1:** Descriptive statistics of the predictor variables.

Predictor Variable	Type/Unit	Change Score Summary (Δ)
WHO-5 total score (T_WHO5)	Continuous (score)	Mean 27.30 (SD 20.04); range −12.00 to 60.00
Chair stand test	Continuous (repetitions)	Mean 2.06 (SD 0.67); range 1.00 to 4.00
Arm curl test	Continuous (repetitions)	Mean 2.08 (SD 3.89); range −8.00 to 10.00
2-Minute Step Test	Continuous (steps)	Mean 11.23 (SD 15.41); range −32.00 to 44.00
Chair sit-and-reach	Continuous (cm)	Mean 0.98 (SD 2.76); range −6.00 to 6.90
Back scratch test	Continuous (cm)	Mean 0.94 (SD 2.78); range −6.00 to 8.00
8-foot up-and-go	Continuous (seconds)	Mean −0.89 (SD 1.54); range −5.30 to 3.40
SF-12 Physical Component Summary (PCS)	Continuous (score)	Mean −1.81 (SD 8.17); range −23.20 to 22.97
SF-12 Mental Component Summary (MCS)	Continuous (score)	Mean −0.98 (SD 7.85); range −25.03 to 19.00
Age group	Categorical	60–64: 152 (33.8%); 65–69: 85 (18.9%); 70–74: 79 (17.6%); 75–79: 60 (13.3%); 80–84: 41 (9.1%); 85–89: 33 (7.3%)
Intervention group	Categorical	Dance: 150 (33.3%); Coordinative: 150 (33.3%); Multimodal: 150 (33.3%)

Change scores (Δ) were computed as the difference between post-intervention and baseline values. For most predictors, positive Δ values represent improvements following the intervention. An exception applies to the 8-foot up-and-go test, for which lower completion times indicate better performance; accordingly, negative Δ values denote functional improvement. In the chair stand test, all observed changes were positive within this sample (Δ ≥ 1), indicating uniform gains in lower-body strength performance.

## Data Availability

The datasets generated and/or analyzed during the current study are not publicly available due to ethical and privacy considerations, but are available from the corresponding author upon reasonable request.

## Abbreviations

The following abbreviations are used in this manuscript:

WHO-5	The World Health Organization Well-Being Index
PCS	Physical Component Summary
MCS	Mental Component Summary
SFT	Senior Fitness Test
SF-12	The Short Form-12 Health Survey
GridSearch	Grid Search Approach
Clasf_Roy	Dependent Variable
AUC	Area Under Curve
ROC	Receiver Operating Characteristic curve
